# Aquaporin-9 in the Brain Inflammatory Response: Evidence from Mice Injected with the Parkinsonogenic Toxin MPP^+^

**DOI:** 10.3390/biom13040588

**Published:** 2023-03-24

**Authors:** Soulmaz Zahl, Nadia Skauli, Katja Stahl, Agnete Prydz, Mina Martine Frey, Erik Dissen, Ole Petter Ottersen, Mahmood Amiry-Moghaddam

**Affiliations:** 1 Laboratory of Molecular Neuroscience, Division of Anatomy, Department of Molecular Medicine, Institute of Basic Medical Sciences, University of Oslo, P.O. Box 1105, Blindern, 0317 Oslo, Norway; 2 Immunobiological Laboratory, Division of Anatomy, Department of Molecular Medicine, Institute of Basic Medical Sciences, University of Oslo, P.O. Box 1105, Blindern, 0317 Oslo, Norway; 3 Karolinska Institutet, 171 77 Stockholm, Sweden

**Keywords:** aquaglyceroporin, AQP9, neuroinflammation, substantia nigra, astrocyte, microglia, flowcytometry

## Abstract

More than 20 years have passed since the first demonstration of Aquaporin-9 (AQP9) in the brain. Yet its precise localization and function in brain tissue remain unresolved. In peripheral tissues, AQP9 is expressed in leukocytes where it is involved in systemic inflammation processes. In this study, we hypothesized that AQP9 plays a proinflammatory role in the brain, analogous to its role in the periphery. We also explored whether *Aqp9* is expressed in microglial cells, which would be supportive of this hypothesis. Our results show that targeted deletion of *Aqp9* significantly suppressed the inflammatory response to the parkinsonian toxin 1-methyl-4-phenylpyridinium (MPP^+^). This toxin induces a strong inflammatory response in brain. After intrastriatal injections of MPP^+^, the increase in transcript levels of proinflammatory genes was less pronounced in AQP9^−/−^ mice compared with wild-type controls. Further, in isolated cell subsets, validated by flow cytometry we demonstrated that Aqp9 transcripts are expressed in microglial cells, albeit at lower concentrations than in astrocytes. The present analysis provides novel insight into the role of AQP9 in the brain and opens new avenues for research in the field of neuroinflammation and chronic neurodegenerative disease.

## 1. Introduction

Aquaporin-9 (AQP9) is a member of the aquaglyceroporin subfamily, with permeability to a wide range of noncharged solutes such as glycerol, urea, monocarboxylates, purines, and pyrimidines in addition to water [1]. More than 20 years have elapsed since AQP9 was identified in the brain [2], yet there are still significant voids in our knowledge as to the localization and function of this aquaporin in brain tissue. Early immunocytochemical analyses identified AQP9 in tanycytes [2], astrocytes, and dopaminergic neurons [3]. Later and more elaborate analyses based on high-resolution immunogold cytochemistry revealed AQP9 in astrocytic plasma membranes and mitochondria in the rat brain [4]. In a more recent study, the expression of AQP9 in the mouse brain, and in particular substantia nigra, was confirmed using *Aqp9* knock-out mice as controls [5]. These antibody-based studies need to be complemented by other methodological approaches to get a more accurate picture of AQP9 localization in the brain. 

As to the functional role of AQP9, there is a rich literature indicating that this aquaporin is involved in peripheral inflammatory responses [6]. AQP9 is expressed by blood leukocytes including monocytes, and AQP9^−/−^ animals are partially resistant to LPS-induced endotoxic shock [7]. Furthermore, inhibition of AQP9 was found to be protective in a mouse model of septic cardiomyopathy [8]. Taken together, available data provide a strong case for the proinflammatory role of AQP9 in peripheral organs. 

We have previously shown that deletion of aquaporin-4 (AQP4), the predominant aquaporin in the central nervous system and a water-selective member of the aquaporin family, significantly reduces the inflammatory response to intrastriatal injections of 1-methyl-4-phenylpyridinium (MPP^+^)—a parkinsonogenic compound that is known to induce a strong inflammatory response [9]. This finding inspired us to use the same experimental model to explore whether AQP9—similar to AQP4 and analogous to the situation in peripheral tissues—plays a proinflammatory role in the brain. The current experimental model is highly relevant from a pathophysiological point of view as intracerebral administration of MPP^+^ is known to cause extensive loss of dopaminergic neurons, mimicking the pathological hallmarks of Parkinson’s disease [10]. 

If the role of AQP9 in the brain is analogous to its role in peripheral tissues, one would expect this aquaporin to be expressed in microglial cells and not only in astrocytes. This is pertinent to the documented presence of AQP9 in blood leukocytes including monocytes, which have the same embryonic precursors as microglia [11,12]. Thus, as a second part of the present study, we re-examined the expression of AQP9 in different types of brain cells following magnetic bead separation and flow cytometry. 

Our findings show that the targeted deletion of *Aqp9* significantly suppressed the inflammatory response after MPP^+^ administration. Specifically, the increase in the transcript levels of proinflammatory genes was less pronounced in *Aqp9^−/−^* mice compared to the wild-type (WT) controls. Secondly, we demonstrate that *Aqp9* transcripts are expressed in microglial cells, albeit at lower concentrations than in astrocytes. The microdistribution of Aqp9 transcripts in substantia nigra was found to be consistent with a predominant expression in astrocytes and microglia. 

## 2. Materials and Methods

### 2.1. Experimental Animals

Adult (2–6 months) mixed gender *Aqp9^−/−^* mice backcrossed with C57BL/6J mice (Jacksons Laboratories), and the littermate WT offspring were used in this study. *Aqp9^−/−^* mice were offspring of *Aqp9* knockout mice generated by Søren Nielsen’s laboratory, University of Aarhus, Denmark [13]. All animals had *ad libitum* access to food and drinking water. For all the analyses, we carried out a statistical comparison between genders where possible. Where statistical analysis wasn’t possible due to the low number of animals for one gender, the analysis was also performed omitting the gender in question. All animal experiments were performed according to the European Council Law on Protection of Laboratory Animals and were approved by The Norwegian Animal Research Authority (NARA) with license FOTS numbers 4012 and 3730. The experiments were in accordance with the European Directive 2010/63/EU.

### 2.2. Stereotaxic Surgery and Microinjection of MPP^+^

WT mice (*n* = 7, including 5 males and 2 females) and *Aqp9*^−/−^ mice (*n* = 7, including 4 males and 3 females) were anesthetized with an intraperitoneal injection of zoletil mixture (Zoletil Forte (250 mg/mL), Rompun (20 mg/mL) and Fentanyl (50 μg/mL); 0.1 mL/10 g) and subjected to the unilateral intrastriatal injection of MPP^+^ (Sigma Aldrich, St. Louis, MO, USA) as described in detail previously [10]. 

Seven days after the operation, animals were decapitated under isoflurane anesthesia. Brains were cut sagittally in two hemispheres (injected and noninjected) and regions of interest, including the midbrain, were dissected out from each hemisphere. The samples were snap frozen in liquid nitrogen and stored at −80 °C.

### 2.3. Microbead-Based Cell Separation of Microglia and Astrocytes

a. Cell separation

WT (*n* = 8, including 6 male and 2 female) and Aqp9^−/−^ (*n* = 8, including 6 male and 2 female) adult mice were deeply anesthetized and transcardially perfused with heparin saline (0.05% heparin (5000 IU/mL) in 0.9% NaCl sterile saline) solution 20 mL/min for 4 min. Brains were dissected out, the cerebellum and olfactory bulb removed and the rest suspended in ice-cold HBSS (#14175129, Gibco, Carlsbad, CA, USA). Brains were mechanically dissociated with sterile scissors in HBSS and further disrupted by pipetting up and down with 1 mL pipette tips until a homogenous suspension of ~1 mm^3^ pieces was achieved. Suspensions were transferred to 15 mL tubes and ice-cold HBSS was added to a final volume of 4 mL, then centrifuged at 300× *g* for 5 min at 4 °C. The supernatant was carefully removed, and the pellet enzymatically dissociated using an Adult Brain Dissociation Kit (130-107-677, Miltenyi Biotec, Bergisch Gladbach, Germany) according to the manufacturer’s instructions. Briefly, preheated (37 °C) Enzyme mix 1 was added to each sample, and incubated under continuous rotation (100 rpm) for 15 min. Enzyme mix 2 was added to each sample, mixed, and incubated at 37 °C under continuous rotation (100 rpm) for 10 min. The cell suspensions were filtered through 70 μm cell strainers and centrifuged at 300× *g* for 10 min at 4 °C.

b. Debris removal

Cell pellets were resuspended in 3.1 mL cold PBS, transferred to a 15 mL tube, and mixed with 0.9 mL cold Debris Removal Solution. The solutions were gently overlaid with 4 mL cold PBS. Tubes were centrifuged at 3000× *g* for 10 min with full acceleration and full brake, and the two top layers containing debris and myelin were removed. Tubes were gently inverted and centrifuged at 1000× *g* for 10 min, followed by the removal of the supernatant.

c. Magnetic labeling of CD11b+ cells

Pellets were resuspended in 90 µL MACS buffer (Miltenyi Biotec). Then, 10 µL of CD11b MicroBeads solution (130-049-601, Miltenyi Biotec) was added, mixed, and incubated at 4 °C for 15 min. Cells were washed by adding 2 mL of MACS buffer and centrifuged at 300× *g* for 10 min. The supernatant was removed and 10 µL were used for cell counting.

d. Magnetic separation of CD11b+ cells

LS columns and a MACS^®^Separator (Miltenyi Biotec) were used for the magnetic separation. The columns were placed in the separator and rinsed with 3 mL of MACS buffer. Cell suspensions labeled with CD11b MicroBeads were applied onto the columns and washed with 3 × 3 mL of the MACS buffer. The total flow through was collected, containing astrocytes and other cells. The columns were removed from the magnet and flushed with 5 mL MACS buffer using a plunger supplied with the column. Then, 10 µL of the CD11b+ cell suspension was used for cell counting.

e. Magnetic labeling of ACSA-2+ cells

The flowthrough was centrifuged at 300× *g* for 10 min. The pellets were resuspended in 80 µL AstroMACS Separation buffer (130-117-336, Miltenyi Biotec) and mixed with 10 µL of FcR Blocking Reagent (130-092-575, Miltenyi Biotec). The solutions were added to 10 µL Anti-ACSA-2 MicroBeads (130-097-679, Miltenyi Biotec) and mixed. The cells were then washed by adding 1 mL of AstroMACS Separation buffer, centrifuged at 300× *g* for 5 min, and the supernatant removed. Up to 10^7^ cells were resuspended in 500 µL of AstroMACS Separation buffer.

f. Magnetic separation of ACSA-2+ cells

The magnetic separation of ACSA-2+ cells was performed similarly to CD11b+ cells, except for replacing the MACS buffer with the AstroMACS Separation buffer. The purity of the cell fractions was increased by repeating the procedure using new columns. Flow through was collected for negative controls.

g. Flow cytometric analysis

There were 25,000–100,000 cells used from the CD11b+, ACSA-2+, and flowthrough fractions. The fractions were transferred to a 96-well plate and centrifuged at 300× *g* for 2 min at 4 °C. The cells were resuspended and double stained with anti-CD11b-FITC (6:100 dilution) and anti-ACSA-2-APC (1.5:100) (Miltenyi Biotec) or irrelevant isotype-matched control antibodies in 50 µL MACS buffer supplemented with 0.05% NaN_3_ and incubated for 15 min in the dark at 4 °C. 

Following antibody staining, the samples were centrifuged at 300× *g* for 2 min and resuspended in 250 µL of MACS buffer for flow cytometry analysis (FACSCanto II, BD Biosciences, Franklin Lakes, NJ, USA). Data were collected from at least 50,000 cells per sample and analyzed with FlowJo software (v10).

### 2.4. Laser Capture Microdissection (LCM)

a. Preparation of LCM samples

WT (*n* = 5, 2 males and 3 females) and Aqp9^−/−^ (*n* = 5, all male) animals were decapitated under isofluorane anesthesia. The brains were dissected out, and a coronal block extending from the optic chiasma to the inferior colliculus was cut and mounted in Optimum Cutting Temperature medium (OCT) (Richard-Allan Scientific™ Neg-50™, Thermo Fisher Scientific, Waltham, MA, USA). Samples were snap- frozen in isopentane cooled on dry ice and stored at −80 °C. Serial coronal sections (20 µm) were cut at −20 °C by cryostat (CryoStar NX70, Thermo Fisher Scientific). Sectioning covered the entire SNpc from Bregma −2.70 to −3.80 [14]. The sections were mounted on pre-chilled polyethylene napthalate membrane slides (1.0 PEN, Zeiss, Jena, Germany) for laser capture microdissection (LCM) or on SuperFrost Plus glass slides (Thermo Fisher Scientific) for immunohistochemistry. All the procedures were performed under RNase-free conditions. As PEN membranes are hydrophobic, slides were treated with UV light at 245nm for 30 min before use.

b. Tyrosine Hydroxylase (TH) immunostaining

Sections were fixed in 4% PFA for 15 min, blocked in 2% H_2_O_2_ (AppliChem) for 5 min, then incubated with mouse anti-TH (1:1000, Chemicon ^®^ Merck KGaA, Darmstadt, Germany) followed by secondary antibody and then a Streptavidin-Biotinylated horseradish peroxidase complex (1:100; GE Healthcare). Sections were further treated in 3, 3′ diaminobenzidine tetrahydrochloride solution (DAB) (Sigma Aldrich) and followed by incubation in DAB solution containing 0.03% H_2_O_2_ for 5 min. Histological images were captured using a 20× objective of a slide scanner (AxioScan, Zeiss).

c. Laser capture microdissection (LCM)

LCM was performed according to photoactivated localization microscopy (PALM) protocols (Zeiss) with some modifications based on other optimization protocols from the literature [15]. PEN membrane slides were dipped in 100% RNase-free ethanol on ice containing dehydration beads (Thermo Fisher Scientific) for 1 min to ensure complete dehydration. LCM was performed with the PALM MicroBeam system (Zeiss). To identify regions of interest, IHC-labeled slides were used as a guideline. The location of SN and VTA were visualized and marked with a microscopic ruler. The substantia nigra pars compacta (SNpc) and reticulata (SNpr), ventral tegmental area (VTA), and hippocampus (CA3 and adjacent stratum radiatum) were cut and catapulted into the adhesive microtube cap (Zeiss) controlled by the PALM RoboSoftware. Samples were kept at −80 °C for further processing. Each PEN slide was processed within 30 min to optimize RNA integrity.

### 2.5. RNA Isolation, cDNA Synthesis, and Quantitative Real Time qPCR

Three different sample sets were processed for RNA isolation, cDNA synthesis, and Quantitative Real Time qPCR. The total RNA was isolated, and concentrations were measured, with an exception for the LCM samples, where the total yield was small and equal amounts of RNA were used for cDNA synthesis. The cDNA was synthesized according to the manufacturer’s protocols. The qPCR reaction was performed in a total volume of 20 μL, containing the Power SYBR Green PCR Master Mix (Applied Biosystems, Waltham, MA, USA), forward and reverse primers (10 µM), and cDNA template. A PCR was performed on the StepOnePlus system (Applied Biosystems) with the thermal cycles as follow: 95 °C for 10 min, followed by 40 cycles at 95 °C for 15 s and 60 °C for 1 min. A standard curve, no reverse transcriptase (NRT), and no template control (NTC) were included in the study. The differences in the RNA isolation and qPCR procedure for each sample set is listed in Table 1. The primers used are listed in Table 2. The expression patterns of several endogenous genes (Table 2) were evaluated using NormFinder and the genes with the most optimal stability values were selected as reference genes [16]. *Ubc* was selected as the normalization gene based on the stability value. Copy numbers were obtained using an absolute quantification calculation, as previously described [17]. To verify a normal distribution, a Shapiro–Wilk test was used. The one-way analysis of variance (ANOVA) post hoc Bonferroni was conducted for data analysis. Data samples that were not normally distributed (*Aqp4* in MPP^+^ samples and *Gad1* in LCM samples), were subjected to log transformation before data analysis by ANOVA. We also performed relative quantification (2−ΔΔCT) for all the data points. Data are presented as mean ± 2SEM (standard error of mean), and *p* < 0.05 was considered to be significant. 

## 3. Results

### 3.1. Genetic Deletion of Aqp9 Attenuates the Increase in the Transcript Levels of Proinflammatory Genes Following Unilateral Intrastriatal MPP^+^ Injections

**Figure 1:**
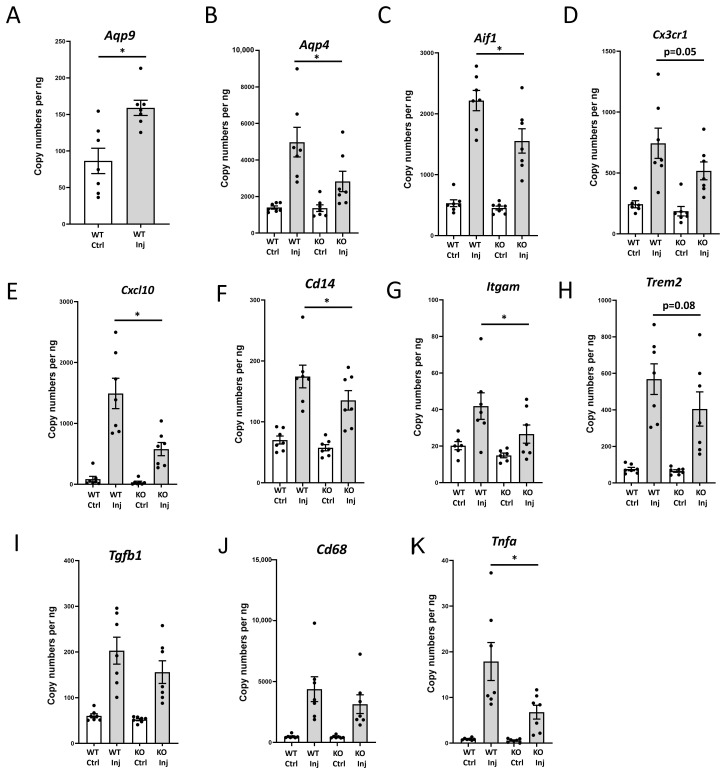
Changes in the RNA levels of *Aqp9* and proinflammatory genes in the ipsilateral midbrain (Inj) compared with the control side (Ctrl) following unilateral intrastriatal injection of MPP^+^ in WT and *Aqp9^−/−^* (KO) mice. (**A**) *Aqp9* expression is significantly increased in the midbrain of the injected side (**B**–**K**) Several inflammatory genes are upregulated in the ipsilateral midbrains of WT animals after MPP^+^ injection compared with the control side, but the upregulation is significantly lower in the midbrains ipsilateral to the injection of the *Aqp9^−/−^* mice. These genes include *Aqp4* (**B**) which is expressed in astrocytes, and the microglial membrane proteins *Aif1* (**C**), *C3xcr1* (**D**), *Cd14* (**F**), and Itgam (**G**), and the inflammatory cytokines *Cxcl10* (**E**) and *Tnfa* (**K**). Transcript levels of the microglial genes *Trem2* (**H**) and *Cd68* (**J**) and the cytokine *Tgfb1* (**I**) were not significantly different in the injected hemisphere of the two genotypes. Bars are mean ± 2SEM, * *p* < 0.05.

Quantitative real time PCR analysis of samples from the midbrains of WT mice subjected to unilateral intrastriatal MPP^+^ injections showed a strong increase in the expression of *Aqp9* in the injected hemisphere (159 ± 15) compared with the control hemisphere (86 ± 10, *p* < 0.0001) (Figure 1A). We then investigated the effect of MPP^+^ on the expression of various inflammation markers in the WT and *Aqp9*^−/−^ mouse midbrains. In WT animals, all markers showed a sizable increase following MPP^+^ injections. Overall, this increase was attenuated in animals with a targeted deletion of *Aqp9,* even though statistical significance was not reached for all genes (Figure 1B–K). 

AQP4 was previously shown to be proinflammatory [9]. After MPP^+^ injections, the transcript levels of *Aqp4* were strongly upregulated in both genotypes, though less so in animals lacking *Aqp9* (WT: 4977 ± 110; *Aqp9*^−/−^: 2825 ± 762, *p* < 0.0001) (Figure 1B). The same pattern was observed for most of the other inflammatory genes in the injected hemispheres, including microglial membrane proteins *Aif1* (WT: 2217 ± 242 vs. *Aqp9*^−/−^: 1554 ± 274, *p* < 0.0001), *C3xcr1* (WT: 743 ± 171 vs. *Aqp9*^−/−^: 517 ± 100, *p* = 0.025), Cd14 (WT: 174 ± 26 vs. *Aqp9*^−/−^: 135 ± 22, *p* = 0.022), and *Itgam* (WT: 42 ± 10 vs. *Aqp9*^−/−^: 27 ± 7, *p* = 0.026), and the inflammatory cytokines *Cxcl10* (WT: 1492 ± 340 vs. *Aqp9*^−/−^: 578 ± 148, *p* < 0.0001) and *Tnfa* (WT: 17.7 ± 6 vs. *Aqp9*^−/−^: 6.7 ± 2.6, *p* < 0.0001). Transcript levels of the microglial genes *Trem2* (WT: 156 ± 116 vs. *Aqp9*^−/−^: 404 ± 130, *p* = 0.067) and *Cd68* (WT: 4376 ± 1413 vs. *Aqp9*^−/−^: 3142 ± 524, *p* = 0.319) and the cytokine *Tgfb1* (WT: 202 ± 41 vs. *Aqp9*^−/−^: 155 ± 34, *p* = 0.106) were not significantly different in the injected hemisphere of the two genotypes. No differences were observed in the transcript levels of the above-mentioned genes in the control hemispheres of the WT and *Aqp9*^−/−^ mice. We also performed relative quantification (2^−ΔΔCT^) for all the data points. The relative quantification showed the same trend and statistical conclusion as the absolute quantification. Analyses of the data from male and female animals showed no gender-dependent differences.

### 3.2. Isolation of Microglia and Astrocytes from the Brains of WT and Aqp9^−/−^ Mice

**Figure 2:**
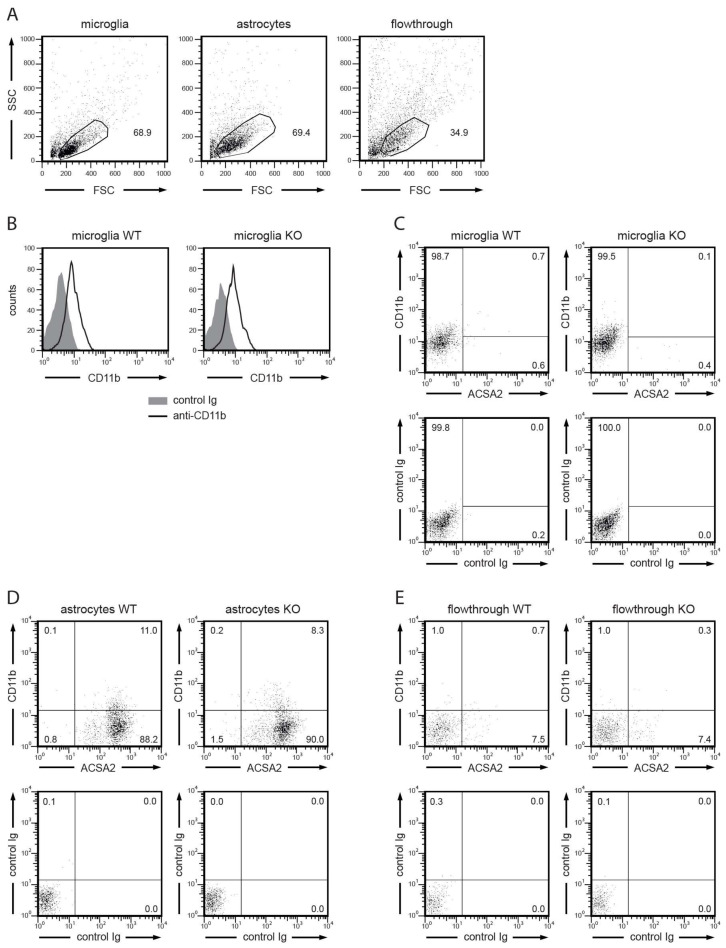
Microglia and astrocyte subsets were isolated from mouse brains, and the purity of the isolates was analyzed by flow cytometry. Gated on viable cells (**A**), the microglia fraction uniformly stained with a FITC-conjugated mAb for CD11b (**B**,**C**) though not with the astrocyte marker ACSA2 (**C**). CD11b staining intensity was low since the cells had been preincubated with a nonlabelled anti-CD11b mAb as part of the purification protocol. Conversely, the astrocyte (**D**) and flowthrough (**E**) fractions were mostly ACSA2^+^CD11b^−^ (with some cells dimly expressing CD11b) or ACSA2^−^CD11b^−^, respectively. Staining with irrelevant control antibodies is shown for comparison.

To resolve whether microglia express *Aqp9*, we isolated microglia and astrocytes from the brains of WT and *Aqp9^−/−^* mice. Successful isolation of the microglia fraction was verified by flow cytometry, demonstrating a >98% pure and uniform CD11b^+^ACSA2^−^ population (Figure 2B,C). Of note, the dim staining with the fluorescence-conjugated CD11b mAb was due to partial blocking resulting from previous staining with unlabeled anti-CD11b mAb as part of the cell isolation procedure. The astrocyte fraction was found to be more than 98% positive for ACSA2. Although the majority of this fraction was ACSA2^+^CD11b^−^, around 10% of the astrocyte fraction dimly coexpressed CD11b with ACSA2. This fraction had not been preincubated with an unlabeled CD11b antibody, indicating that the observed CD11b surface expression was naturally low. The flow-through fraction did not stain significantly with CD11b, nor with ACSA2. 

### 3.3. Quantitative Real-Time PCR of Isolated Cells Shows Expression of Aqp9 in Microglia

**Figure 3:**
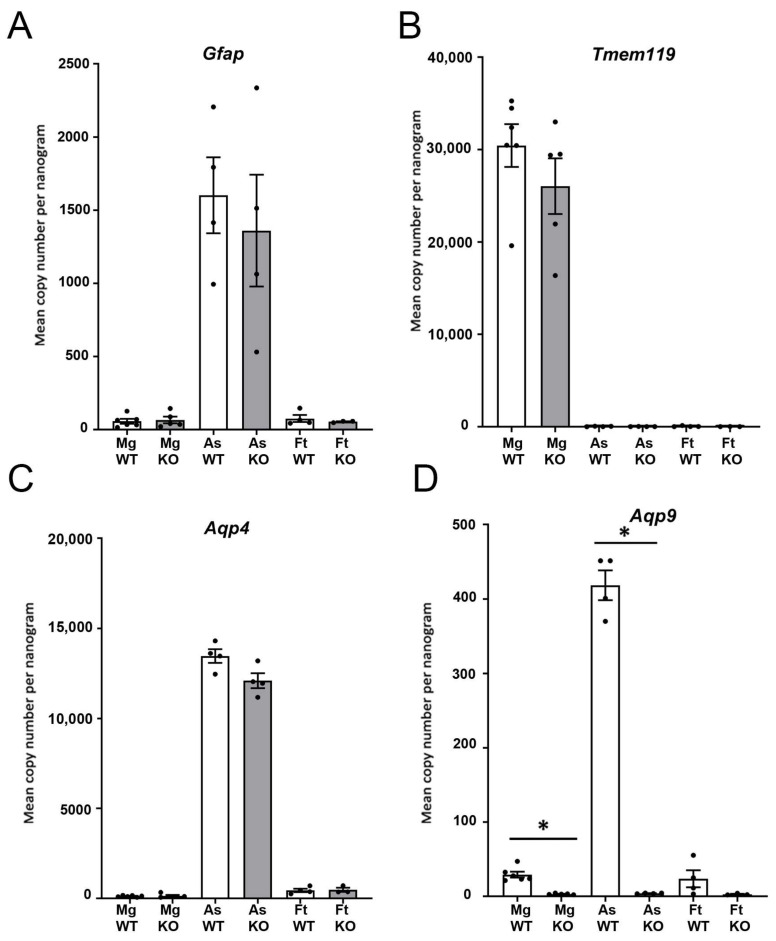
Gene expression in magnetic bead-separated cells from WT and *Aqp9^−/−^* mouse brains (**A**) The astrocyte marker *Gfap* is highly expressed in the astrocyte fraction in both genotypes. (**B**) The microglial marker *Tmem119* is only expressed in the microglial cell population of both genotypes. (**C**) *Aqp4* is prominently expressed in the astrocyte fraction in both genotypes. (**D**) *Aqp9* expression is seen in the WT microglia and astrocytes, whereas *Aqp9^−/−^* cell populations show no expression. Bars are mean ± 2SEM, * *p* < 0.05.

The purity of the cell fractions was evaluated with quantitative PCR. The vast majority of cells identified by the ACSA2 astrocyte marker contained *Gfap* mRNA (Figure 3A). This was true for WT as well as *Aqp9^−/−^* mice. Other cell populations (microglia and flow through) showed very low copy numbers for *Gfap* mRNA attesting to the purity of the cell populations. Microglia were also clearly separated from other cell populations (Figure 3B). The microglial marker *Tmem119* [18], showed 30,000 times higher expression in the microglia than in the astrocytes (30,443 ± 4633 compared to 34 ± 25 and 53 ± 56 in the astrocytes and flowthrough samples, respectively). 

Supporting previous studies, *Aqp4* expression was restricted to the astrocyte fraction (Figure 3C). *Aqp4* showed 12,000 times higher expression in the astrocytes compared to the other cell types (13,465 ± 760 compared to 123 ± 34 and 448 ± 191 in microglia and flowthrough samples, respectively). Similar to *Aqp4* mRNA, *Aqp9* mRNA was clearly most abundant in the astrocyte fraction (Figure 3D). However, a significant signal was obtained also from the microglia fraction. The qPCR results revealed that the mRNA expression of *Aqp9* is around 10 times higher (*p* < 0.001) in the astrocytes (418 ± 40) compared with the microglia (30 ± 7.8). This indicates that *Aqp9* is expressed in all microglia at a very low level or expressed at a higher level in a subpopulation of microglia. 

### 3.4. The mRNA Levels of Aqp9 in Midbrain Subregions

We then analyzed the expression of *Aqp9* in the substantia nigra pars compacta (SNpc), substantia nigra pars reticulata (SNpr), and the ventral tegmental area (VTA) isolated by laser capture. The hippocampus was used as a reference region. As expected, the dopamine transporter *Slc6a3* was highly expressed in fractions containing the substantia nigra pars compacta (37,658 ± 8660) and VTA (30,435 ± 5256), while its expression was much lower in the SNpr (4300 ± 2908) and the hippocampus (124 ± 117). The pars reticulata contained an overabundance of astrocytic and microglia markers, compared with other regions. The expression of *Gfap* as an astrocyte marker was significantly higher in SNpr than SNpc (4737 ± 1770 and 811 ± 160, *p* < 0.001). The expression of *Tmem119* as a microglia marker was significantly higher in SNpr than SNpc (337 ± 104 and 59.4 ± 30 *p* < 0.001). Surprisingly, the expression of *Gfap* and *Tmem119* was significantly higher in *Aqp9*^−/−^ compared with the WT in SNpr (*Gfap*: *Aqp9^−/−^* 8663 ± 2516 compared to WT 4737 ± 1770 *p* = 0.001; and *Tmem119*: Aqp9^−/−^ 683 ± 189 compared with WT 337 ± 104 *p* < 0.001), which could be due to a slight astrogliosis and microgliosis in *Aqp9^−/−^* animals. As expected, *Gad1* was more strongly expressed in the pars reticulata than in other regions, reflecting a prevalence of GABA-ergic cells. *Gad1* expression was higher in SNpr with an expression level of 56,177 ± 20,196, compared to 36,289 ± 8495 in SNpc, although there was no statistically significant difference between the two subregions (*p* = 0.55), possibly due to a slight admixture of SNpr into the SNpc samples.

**Figure 4:**
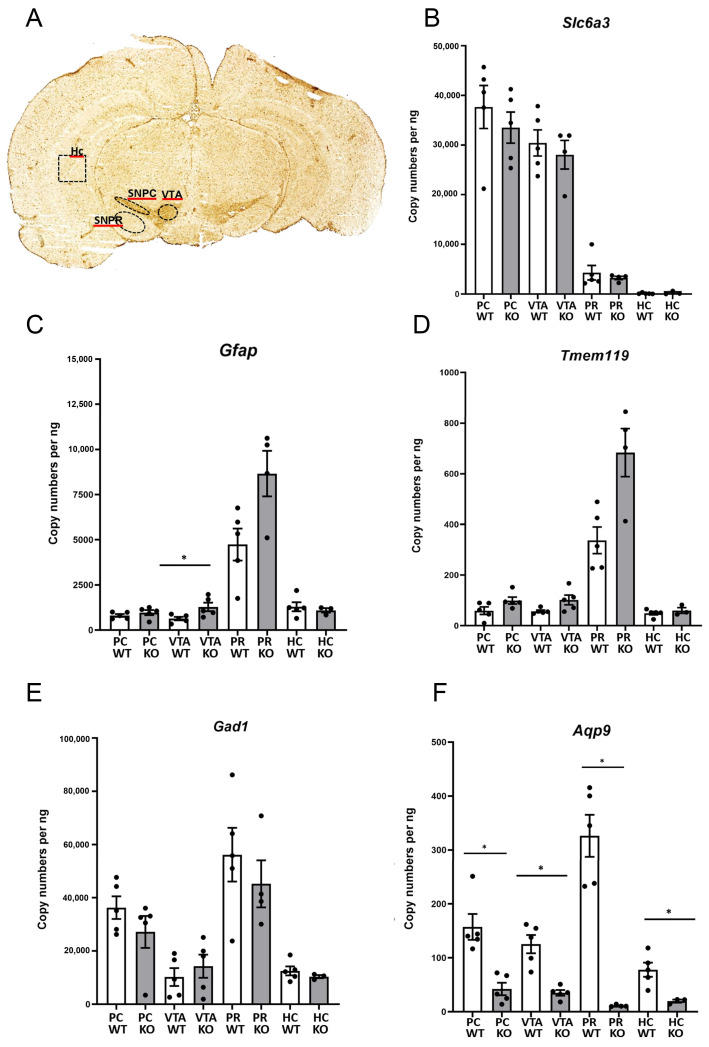
Regions selected for laser capture and expression of transcripts related to neuroinflammation in the dissected regions. (**A**) Overview of regions selected for laser capture on a scan of a section immunostained with an antibody to the dopaminergic cell marker tyrosine hydroxylase (TH). (**B**) *Slc6a3*, the transcript for the dopamine transport DAT, is highly expressed in the pars compacta and VTA, with no significant difference between genotypes. (**C**) The astrocyte marker *Gfap* is highly expressed in the pars reticulata, with a statistically significant higher expression in *Aqp9^−/−^* animals in the VTA. (**D**) The microglial marker *Tmem119* is highly expressed in the pars reticulate. (**E**) *Gad1* is expressed in all the investigated regions, with no significant difference between genotypes. (**F**) *Aqp9* is expressed in all investigated regions of the WT mice, with the highest expression level in the pars reticulata. Bars are mean ± 2SEM, * *p* < 0.05.

*Aqp9* transcripts were similarly more plentiful in the pars reticulata. This is in line with the richness of the astrocytes and microglia in this region. For *Aqp9*, the mRNA copy numbers were 326.4 ± 77.8 in SNpr, 157.4 ± 47.7 in SNpc, 125.6 ± 33.8 in VTA, and 78.2 ± 26.1 in the hippocampus (Figure 4). 

## 4. Discussion

Surprisingly little is known about the localization and function of AQP9 in the brain. Interest has primarily been devoted to the other brain aquaporins, not least AQP4 which is by far the most prevalent brain aquaporin. The brain also contains AQP1, which plays distinctive roles in the choroid plexus, and AQP11 which seems to be very weakly expressed and apparently only at the transcript level [19,20,21].

AQP4 and AQP1 are classical aquaporins, implying that they are highly specific for water. The strict substrate specificity is explained by the structure of the pore channel whose dimension and lining allow water to pass while restricting the passage of ions and larger molecules [20]. AQP9, by comparison, is endowed with a wider pore that mediates the flux of a number of larger organic molecules [1,6]. This property makes AQP9 an interesting focus of study not least in the realm of metabolite transport. 

Research on AQP9 has been hampered by its relatively low expression in the brain compared to the blood and peripheral organs (such as the liver) where its role has been easier to resolve [22,23,24]. Methodological obstacles have also delayed progress since specific and high avidity antibodies have been difficult to come by. Renewed interest in brain AQP9 is now kindled by the recent discovery that *Aqp9* deletion protects against MPP^+^-induced cell death in a mouse model of Parkinson´s disease [10].

In the present study, we used methodological approaches complementary to those employed in previous analyses to demonstrate that *Aqp9* is expressed not only in brain astrocytes but also in microglial cells and that *Aqp9* deletion significantly curbs the inflammatory response following intrastriatal MPP^+^ injections. This is the first demonstration of an aquaporin in microglial cells and the first evidence of a coupling between AQP9 and inflammatory responses in the brain. The role of AQP9 in microglia ties in with previous studies showing AQP9 expression in leukocytes and inflammatory responses in peripheral tissues [25]. 

Our conclusion—that AQP9 deletion suppresses the inflammatory response—was based on an analysis of specific proinflammatory genes. Notably, *Aqp9* deletion dampened the MPP^+^-induced increase in the expression of the gene encoding TNFα, an inflammatory cytokine secreted from several cell types including the microglia [26]. In addition to standard inflammatory markers, we also analyzed the expression of *Aqp4*. The expression of this gene was increased following MPP^+^ injections but much more so in wild-type animals than in the *Aqp9^−/−^* mice. In other words, *Aqp4* expression changed in parallel with the expression of established proinflammatory genes, in line with our previous observations [9]. 

Further studies are needed to unravel the mechanistic underpinning of the present observations. The downregulation of microglial inflammation markers following *Aqp9* deletion might be a direct consequence of removing the microglial pool of AQP9. Alternatively, the dampening of the inflammatory response might be an indirect effect, caused by the loss of AQP9 from astrocytes that normally interact with microglial cells. That such indirect effects might be at play is suggested by recent observation in AQP4 knock-out mice that, similarly to *Aqp9^−/−^* mice, show a blunted inflammatory response following MPP^+^ injections. Notably, AQP4 is strictly localized to the astroglial cells [27]. This implies that a loss of this aquaporin should not affect microglia directly, but more likely it exerts an effect through modulating the release of astroglial signaling molecules that normally regulate microglial function. 

The function of AQP9 in the brain has proven to be elusive. Here we hypothesized that AQP9 plays a proinflammatory role in the brain, mimicking its well-documented role as a proinflammatory agent in peripheral organs [6,28,29]. In line with this hypothesis, we found that deletion of *Aqp9* curbed the increase of inflammatory markers in the midbrain after intrastriatal injections of the parkinsonogenic toxin MPP^+^. 

Mice subjected to intrastriatal MPP^+^ injections were chosen as a model since these mice display a clear and spatially constrained inflammatory response in the substantia nigra [9]. This study was focused on the acute phase (one-week postinjection) and was not designed to explore the long-term effects of the inflammatory response. It is relevant, though, that chronic inflammation is considered a hallmark of Parkinson’s disease and likely of pathophysiological significance [30,31]. Thus, the mechanisms addressed in the present study deserve to be followed up in more chronic models of the disease. 

Previous studies of AQP9 have been challenged by its low expression level in the brain compared to the liver and other peripheral organs [5]. The present study is no exception. *Aqp9* was found to be weakly expressed compared with Aqp4, and this complicates the analysis. It is notable, however, that the effects of *Aqp9* deletion on the inflammatory responses to MPP^+^ treatment are robust. When it comes to *Aqp9* expression in microglia, it must be emphasized that for flow cytometry, the microglia were obtained from the whole brain, thus obscuring any regional differences in *Aqp9* expression. The possibility exists that nigral microglia are enriched with AQP9 compared with microglia from the other regions. This would be in line with the present laser capture analysis. Unfortunately, the antibodies currently available are not of sufficient avidity and specificity to resolve this issue. 

In conclusion, the present study confirms that *Aqp9* is expressed in astrocytes and is the first to show that this aquaporin also occurs in microglia. Whether its proinflammatory effect depends on the astrocytic pool, the microglial pool, or both needs to be resolved by forthcoming studies. Similarly, the detailed mechanisms involved require further scrutiny. In regard to peripheral organs, it has been suggested that the proinflammatory role of AQP9 may be coupled to its ability to take up H_2_O_2_ [7]. A similar mechanism may apply in the brain. Another possibility is that AQP9 facilitates microglial migration, which is an essential part of the inflammatory response. Since AQP9 is permeable to a wide range of molecules including MPP^+^ [10], one cannot rule out the possibility of an activating effect of MPP^+^ on astrocytes and microglia. Regardless of mechanism involved, the present analysis provides a novel insight into the role of AQP9 in the brain and opens new avenues for research in the fields of neuroinflammation and chronic neurodegenerative disease. 

## Figures and Tables

**Table 1:** RNA isolation, cDNA synthesis, and qPCR.

	MPP^+^ Tissues	Separated Brain Cells	LCM
RNA isolation kit	RNeasy^®^ Mini Kit (QIAGEN, Hilden, Germany)	RNeasy^®^ Plus Micro Kit (QIAGEN)	RNeasy^®^ Plus Micro Kit (QIAGEN)
cDNA kit	200ng RNA input GoScript Reverse Transcription System (Promega, Madison, WI, USA)	100ng RNA input GoScript Reverse Transcription System (Promega)	5 μL RNA input Sensiscript^®^ Reverse Transcription Kit (QIAGEN)
Total cDNA template in qPCR	1 ng	2.5 ng	5 μL
Evaluated endogenous reference genes	*Ubc*, *Actb*, *Pgk1*, *H2afz*	*Ubc*, *H2afz*, *Hsp8a*	*Ubc*, *H2afz*, *Hsp8a*

**Table 2:** Primers used for RT-qPCR analysis.

Gene Name	Forward Primer	Reverse Primer
*Aqp9*	TTGCAACGGCAGTTGTGATG	CAAAAGACACCGCTGGGTTG
*Aqp4*	TTTGGACCCGCAGTTATCAT	GTTGTCCTCCACCTCCATGT
*Aif1*	CTGCCAGCCTAAGACAACCA	GGAATTGCTTGTTGATCCCCT
*Cx3cr1*	TGCTCAGGACCTCACCATGTC	CTCAAGGCCAGGTTCAGGAG
*Cxcl10*	ATGACGGGCCAGTGAGAATG	TCGTGGCAATGATCTCAACAC
*Cd14*	CAGAGAACACCACCGCTGTA	CACGCTCCATGGTCGGTAGA
*Itgam*	TGCGCGAAGGAGATATCCAG	GCCTGCGTGTGTTGTTCTTT
*Trem2*	CTGGAGGACCCTCTAGATGAC	CCACAGGATGAAACCTGCCT
*Tgfb1*	AATTCCTGGCGTTACCTTGG	AGTGAGCGCTGAATCGAAAG
*Cd68*	AGGACCGCTTATAGCCCAAG	TCATCGTGAAGGATGGCAGG
*Tnfa*	GGATGAGAAGTTCCCAAATGGC	ACTTGGTGGTTTGCTACGAC
*Gad1*	CAGTCACCTGGAACCCTCAC	CACGGTGCCCTTTGCTTTC
*Gfap*	GCACTCAATACGAGGCAGTG	GCTCTAGGGACTCGTTCGTG
*Tmem119*	CACCCAGAGCTGGTTCCATA	GTGACACAGAGTAGGCCACC

## Data Availability

Not applicable.
